# Tear fluid database: a reference website for tear fluid proteomics

**DOI:** 10.1093/database/baaf091

**Published:** 2026-01-16

**Authors:** Drew Mayernik, Saleh Ahmed, Eliza Williams, Tae Jin Lee, Amy Estes, Pamela Martin, Wenbo Zhi, Vishal Jhanji, Shruti Sharma, Ashok Sharma

**Affiliations:** Center for Biotechnology and Genomic Medicine, Medical College of Georgia, Augusta University, 1120 15th Street, Augusta, GA 30912, USA; Center for Biotechnology and Genomic Medicine, Medical College of Georgia, Augusta University, 1120 15th Street, Augusta, GA 30912, USA; Center for Biotechnology and Genomic Medicine, Medical College of Georgia, Augusta University, 1120 15th Street, Augusta, GA 30912, USA; Center for Biotechnology and Genomic Medicine, Medical College of Georgia, Augusta University, 1120 15th Street, Augusta, GA 30912, USA; Department of Ophthalmology, Medical College of Georgia, Augusta University, 1120 15th Street, Augusta, GA 30912, USA; Department of Ophthalmology, Medical College of Georgia, Augusta University, 1120 15th Street, Augusta, GA 30912, USA; Center for Biotechnology and Genomic Medicine, Medical College of Georgia, Augusta University, 1120 15th Street, Augusta, GA 30912, USA; Department of Ophthalmology, University of Pittsburgh School of Medicine, 1622 Locust Street, Pittsburgh, PA 15219, United States; Center for Biotechnology and Genomic Medicine, Medical College of Georgia, Augusta University, 1120 15th Street, Augusta, GA 30912, USA; Department of Ophthalmology, Medical College of Georgia, Augusta University, 1120 15th Street, Augusta, GA 30912, USA; Center for Biotechnology and Genomic Medicine, Medical College of Georgia, Augusta University, 1120 15th Street, Augusta, GA 30912, USA; Department of Ophthalmology, Medical College of Georgia, Augusta University, 1120 15th Street, Augusta, GA 30912, USA

## Abstract

Tear fluid is a clinically accessible, minimally invasive biofluid with a complex and dynamic proteome. Molecular alterations in tear composition have been linked to a broad spectrum of ocular and systemic diseases; however, the small volume of tear samples presents substantial challenges for obtaining high-quality proteomic data. To overcome this limitation, we developed a highly sensitive mass spectrometry workflow capable of identifying more than 1,000 proteins from individual tear samples. Applying this workflow to a large and diverse cohort, we generated a representative and comprehensive profile of the human tear fluid proteome and established reference abundance ranges for proteins commonly detected in tear fluid. In parallel with protein quantification, we collected detailed clinical annotations for each participant. As the database continues to grow, these analyses will increasingly support the identification of disease-associated proteomic signatures, deepen our understanding of underlying biological mechanisms, and accelerate the discovery of clinically relevant biomarkers. To make these data broadly accessible, we created a user-friendly website for exploring protein measurements alongside associated clinical metadata. The current release includes proteomic profiles from 74 human tear samples, encompassing 2,134 unique proteins. The *TearFluid Database* serves as a foundational resource for biomarker discovery, comparative proteomics, and systems-level investigations in tear biology, offering the scientific community a robust and expandable platform for advancing tear fluid proteomics research.

Database URL: https://tearfluid.org/

## Introduction

The tear fluid is a complex, multilayered biofluid produced through the coordinated secretions of the lacrimal glands (aqueous layer), meibomian glands (lipid layer), and the goblet cells of the conjunctiva (mucin layer) [1–5]. Tear fluid plays essential roles in protecting the eye from environmental stressors, maintaining ocular surface moisture, providing immune defence, and supplying nutrients to the corneal epithelium [2, 6, 7]. Proteins within the tear film contribute to several essential biological functions, including immunological defence [8], oxidative homeostasis [9], and epithelial cell proliferation [10]. Due to its molecular complexity [11], minimally invasive collection, and proximity to the ocular environment, tear fluid has emerged as a powerful tool for investigating the molecular pathophysiology of both ocular and systemic diseases [12–26]. Proteomic variations in tear fluid have been associated with multiple ocular and systemic pathologies, including Parkinson’s disease and other neurodegenerative conditions [13, 14], glaucoma [19, 27, 28], dry eye disease [17, 18, 29], and breast cancer [30].

Analysing the tear proteome is technically challenging due to the small sample volume and the wide dynamic range of protein concentrations. High-resolution technologies, such as mass spectrometry, have emerged as powerful tools for gaining molecular insights from the tear fluid. We recently optimized a liquid chromatography-tandem mass spectrometry (LC-MS/MS) workflow for tear proteomics, enhancing sensitivity and reproducibility to enable the reliable detection of more than 1,000 proteins. Despite these advances, a comprehensive reference database of tear fluid protein levels has yet to be established. Existing datasets are limited in size, scope, standardization, and resolution, with many focusing on targeted panels or subsets of proteins. Reported numbers of identified tear fluid proteins range widely from a few hundred to over a thousand depending on methodology, sample preparation, and instrumentation used. Currently, no openly accessible resource catalogues the full range of proteins detectable in human tear fluid, along with associated clinical metadata. A centralized tear proteome database is therefore essential to provide consistent reference abundance ranges, facilitate cross-study comparisons, and support the identification of disease-related fluctuations. The *TearFluid Database* addresses this unmet need by providing a curated, large-scale, and publicly accessible resource that serves as an expandable platform for centralizing molecular tear data and supporting research in tear fluid biology.

## Materials and methods

### Human subjects and sample collection

Participants in this study were recruited from patients aged 21–90 who were undergoing routine eye examinations at the Department of Ophthalmology, Augusta University. Pregnant individuals were excluded to minimize the influence of potential confounding variables related to hormonal changes. The study was approved by the Institutional Review Board of Augusta University (IRB #2034537), and written informed consent was obtained from all participants.

Tear samples were collected using TearFlo Schirmer strips (09006, LiteSource Medical, Louisville, KY, USA) without topical anaesthesia or any external stimulation, and the wetting length was recorded after 5 minutes. Samples collected from contralateral eyes were not pooled, and each eye was analysed separately. Following collection, samples were immediately transferred to 2 ml cryovials and stored at −80°C until further analysis.

Participants also completed the Ocular Surface Disease Index (OSDI-12), a validated questionnaire widely used in clinical and research settings to assess symptoms of dry eye disease and ocular surface discomfort. During the visit, the physician recorded several clinical parameters, including tear breakup time, corneal and conjunctival staining, lid margin condition, meibum quality, and meibomian gland expressibility. A chart review was performed after sample collection to obtain demographic and additional clinical information for each participant.

### Protein extraction and enzymatic digestion

The in-strip digestion method was used to extract and digest the proteins from Schirmer’s strip [15]. Our optimized in-strip digestion and peptide recovery method minimizes sample loss. In this method, Schirmer strips were first subjected to lyophilization for 30 min and subsequently cut into dimensions of 5.0 mm × 2.5 mm. To denature the proteins, 120 µl of 8 M urea in 50 mM Tris–HCl (pH 8) was added to the tube containing the pieces of strips and incubated in an orbital shaker at 60°C for 30 min.

Samples were reduced with 0.6 µl of 10 mM dithiothreitol and alkylated with 10 µl of 55 mM iodoacetamide, followed by incubation on an orbital shaker for 30 min at room temperature in the dark. The pH of each sample was adjusted and maintained between 7 and 9 using 0–14 pH indicator strips (#13640516, Thermo Fisher Scientific) prior to overnight digestion at 37°C with proteolytic trypsin (#90057, Thermo Fisher Scientific) at a 1:20 (w/w) trypsin-to-protein ratio. After digestion, peptide concentrations were quantified using the Pierce Quantitative Colorimetric Peptide Assay (23275, Thermo Fisher Scientific). An equal amount of peptides from each sample was further purified using C18 spin columns, and the purified eluates were lyophilized overnight to obtain peptide powders. The dried peptides were then reconstituted in 80 µl of equilibrium buffer (2% acetonitrile in 0.1% formic acid) for LC-MS/MS analysis.

### Liquid chromatography-tandem mass spectrometry analysis

LC-MS/MS analysis was conducted using an Orbitrap Fusion Tribrid mass spectrometer coupled with an Ultimate 3000 nano-UPLC system (Thermo Fisher Scientific, Waltham, MA, USA) in data-independent acquisition (DIA) mode. The method described in Ahmed *et al*. [31] was followed for the DIA LC-MS/MS analysis. Two microlitres of reconstituted peptides were loaded and rinsed on a PepMap100 C18 trap column (5 µm, 0.3 × 5.0 mm; Thermo Fisher Scientific, Waltham, MA, USA) at a flow rate of 20 µl/min. The LC gradient was composed of buffer A (0.1% formic acid in LC-grade water) and buffer B (100% acetonitrile containing 0.1% formic acid in LC-grade water).

Peptides were separated on a PepMap100 RSLC C18 column (2.0 µm, 75 µm × 150 mm; Thermo Fisher Scientific) using a 100-min multistep gradient at a flow rate of 300 nl/min and a column temperature of 40°C. The instrument was operated in positive ion mode using a nano-electrospray ionization source, with a spray voltage of 2000 V and an ion transfer tube temperature of 300°C.

An Orbitrap mass analyser was used to perform the precursor mass scan at a resolution of 60 000 with a maximum injection time of 58 ms, covering an *m*/*z* range of 350–1550. Subsequently, 40 MS2 scans were performed across an *m*/*z* range of 400–1200. Fragmentation of precursor ions was performed using higher-energy collisional dissociation at a normalized collision energy of 30%. The isolation window was set to 20 *m*/*z*, with a cycle time of 3 s.

### Protein identification and quantification

Raw mass spectrometry data were processed using DIA-NN (Data-Independent Acquisition by Neural Networks), version 2.0.2 [32]. An *in silico* DIA-NN–predicted spectral library was generated from the 2024 human UniProtKB/Swiss-Prot database (20 412 entries) [33] and used to analyse the raw data with a mass accuracy of 10 ppm and a 1% false discovery rate (FDR). DIA-NN parameters included trypsin/P as the protease, allowance for one missed cleavage, excision of the N-terminal methionine, carbamidomethylation of cysteine, peptide lengths of 7–30 amino acids, a precursor charge range of 1–4, gene-level protein inference, and the Quant UMS (high-precision) measurement approach. Additionally, the options ‘No shared spectra’, ‘Heuristic protein inference’, and ‘MBR’ were enabled. Finally, a comprehensive .parquet report was generated, containing quantification values in the form of peak intensities (MaxLFQ) for each identified protein, along with various confidence metrics. Peak intensity values serve as a semiquantitative metric, allowing us to assess the relative abundance of proteins within the tear fluid proteome.

### Database and website development

**Figure 1.**
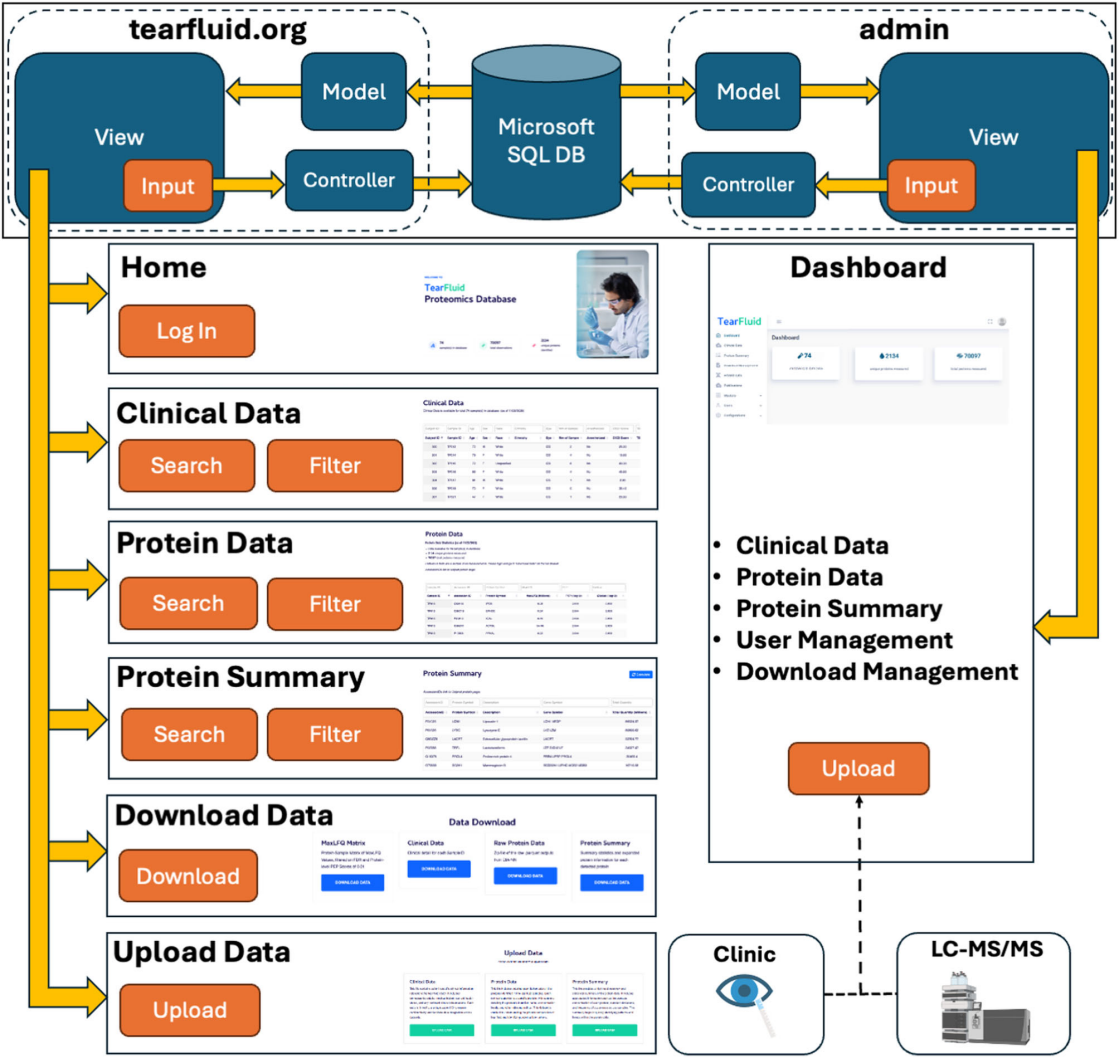
Overview of the TearFluid platform architecture. The top panel illustrates the model–view–controller (MVC) architecture of the TearFluid Database. Controllers query the Microsoft SQL database, process the data as models, and present them to users through views. Each page includes a set of interactive inputs that communicate with controllers to enable data querying. The Admin Dashboard provides a backend interface for developers to curate and manage site data.

The TearFluid Database interactive web application was developed using ASP.NET for backend design and JavaScript for front-end functionality. The web application follows a model–view–controller (MVC) architectural pattern (Fig. 1), a modular design that separates key software components by function to streamline future development. A front-end interface was developed to enable querying, exploration, and interaction with proteomic data. Meaningful user experience (UX) features were incorporated into the front-end design, including data search and filtering capabilities. The backend is implemented in C#, providing functionality for data querying, table generation, and database expansion. A web interface was developed to enable uploading and downloading of data tables, with options to export them to Excel. An admin panel was built alongside the public-facing website to support developer updates and provide an efficient process for expanding the database with new data.

**Figure 2.**
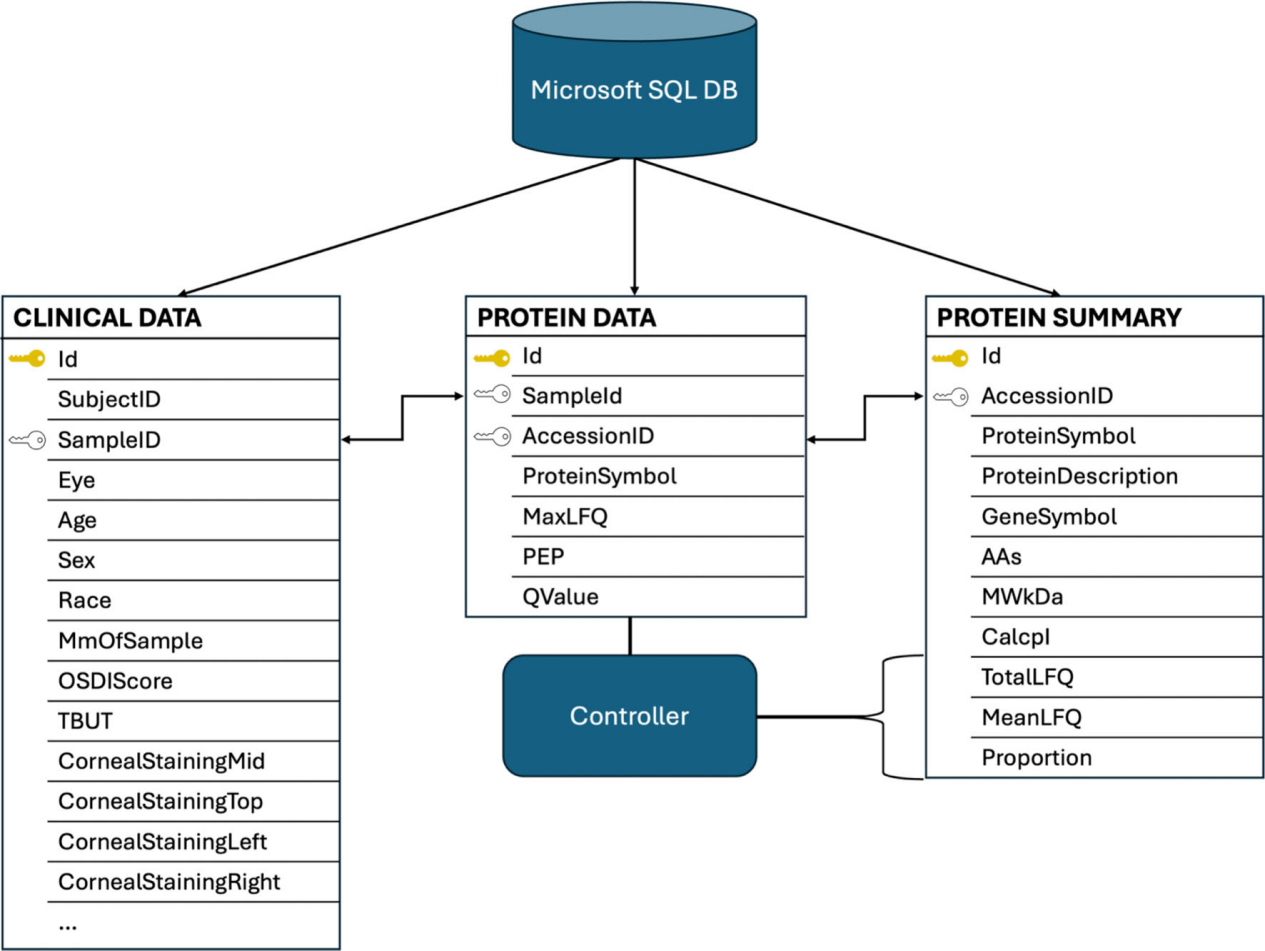
Overview of the TearFluid database schema. The relational design of the database highlights interoperability among tables. The ‘ID’ fields represent hidden, unique server-side identifiers for each record. The ‘Sample ID’ and ‘Accession ID’ fields serve as foreign keys that enable integration across datasets. An example of this coordination is shown, where the backend controller analyses the Protein Data Table to generate the ‘TotalLFQ’, ‘MeanLFQ’, and ‘Proportion’ fields in the Protein Summary Table.

Microsoft SQL server is used for data storage and retrieval. A relational database was designed to support three integrated data modules, enabling exploration of the dataset from multiple perspectives. The database consists of three integrated data tables: Protein Data, Clinical Data, and Protein Summary, each offering a distinct view of the data collected from human tear fluid samples (Fig. 2). The relational and interoperative structure of the database allows for analysis across data modalities and numerous clinical parameters, enhancing the capabilities to derive comparative proteomic insights from the dataset.

## Results

The TearFluid Database is an openly accessible online resource designed to advance tear proteomics research by enabling efficient exploration and integration of clinical and proteomic data. It provides a user-friendly interface and a centralized platform that enables users to navigate, query, and download high-resolution tear proteomic data together with the associated clinical characteristics.

**Figure 3.**
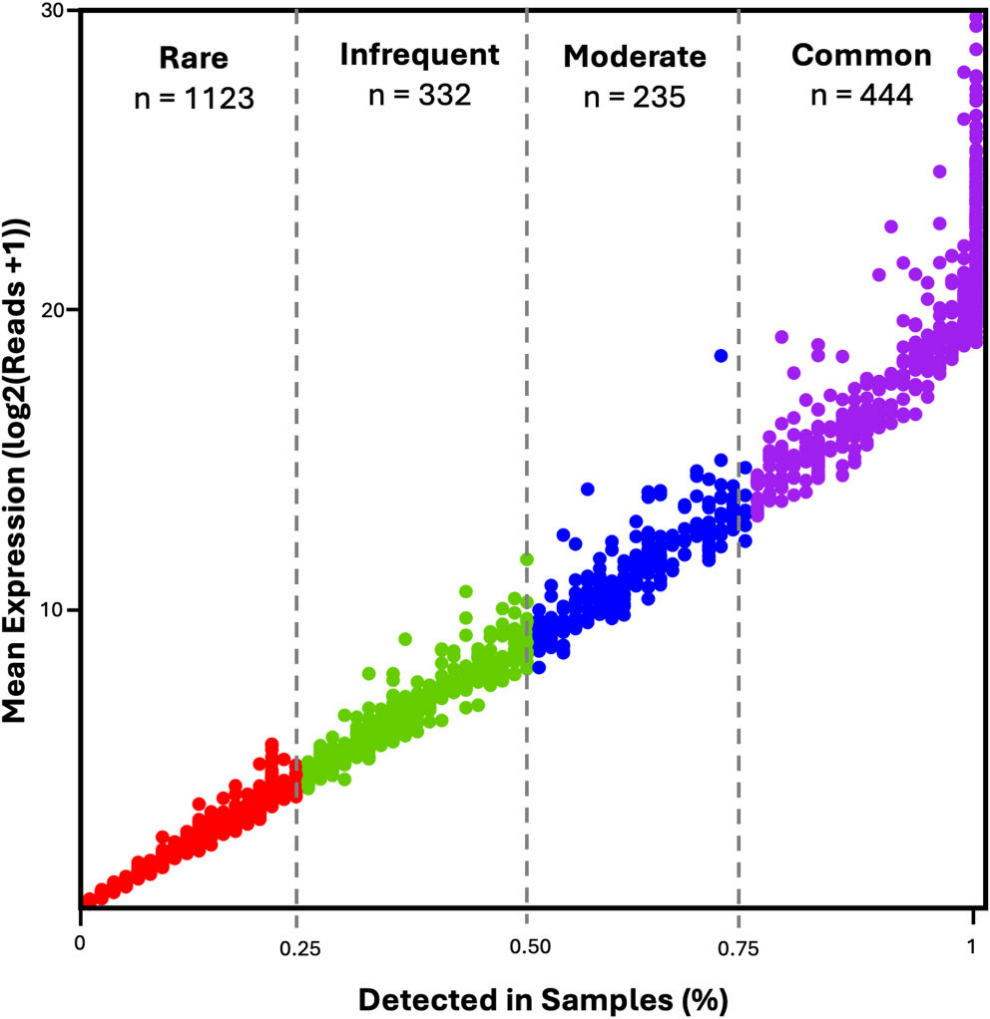
Overview of proteomic expression across samples. Proteins passing a QValue threshold of 0.01 are included (*n* = 2134). Mean log-transformed MaxLFQ expression is plotted against the proportion of samples in which each protein is detected. Proteins are categorized into four abundance classes: Rare (<25% of samples, *n* = 1123), Infrequent (>25% and <50%, *n* = 332), Moderate (>50% and <75%, *n* = 235), and Common (>75%, *n* = 444).

The current release includes samples from 74 unique subjects, representing a diverse population in terms of age, sex, and race (Table 1). A total of 2134 unique proteins were identified across all samples using DIA mass spectrometry (Fig. 3), yielding one of the most comprehensive publicly available tear proteome datasets to date.

**Table 1.** Demographic characteristics of the 74 study subjects.

Variable	Value
Age (mean ± SD)	68.6 ± 10.8 years
Sex (F/M)	54/20
Race (White/Black/Others)	57/14/3

### Protein data

**Figure 4.**
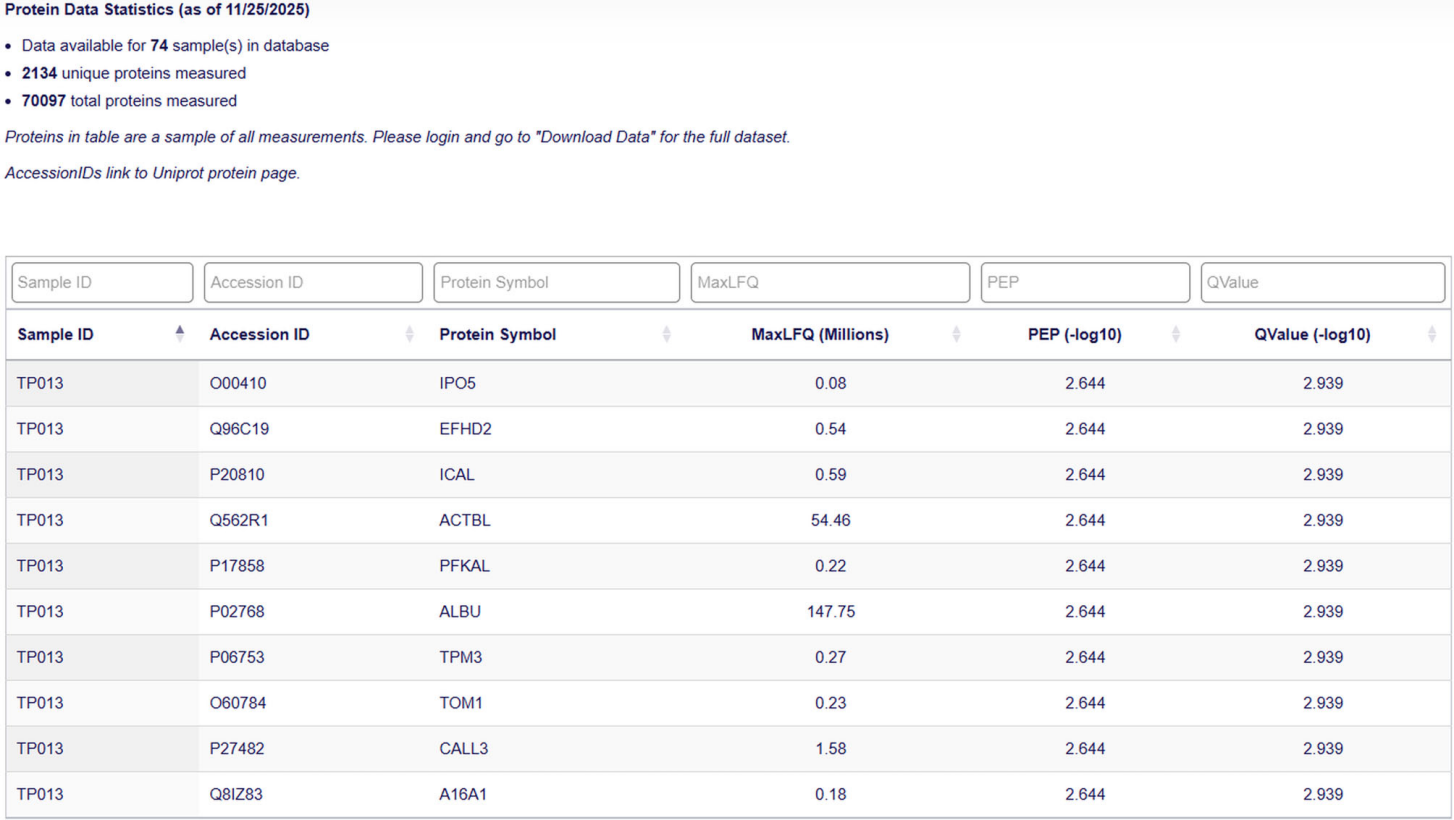
Protein data page. The Protein Data Table displays Sample ID, Accession ID, Protein Symbol, MaxLFQ value, Posterior Error Probability (PEP), and QValue for each protein-sample pair.

The Protein Data Table serves as the foundation of the database, housing all protein group-level mass spectrometry outputs for each tear fluid sample (Fig. 4). Each record in the table is uniquely defined by the combination of the ‘Sample ID’ and ‘Protein Name’ fields. This structure ensures both uniqueness and interoperability with related records in the Clinical Data and Protein Summary Tables. The protein groups were determined using DIA-NN, which identifies proteins based on peptide-level spectral data. The leading protein (i.e. the first protein in the group as defined by DIA-NN) is reported for each protein group.

The proteomic data are quantified using the MaxLFQ score. MaxLFQ is a label-free quantification (LFQ) algorithm that estimates relative protein abundance by integrating peptide ion intensities across samples, providing a robust, semiquantitative measure. MaxLFQ is designed to maximize precision and comparability across runs and is calculated at the protein group level [34]. This score serves as the primary metric for relative protein quantification in the database and supports a broad range of comparative analyses.

In addition to abundance, two metrics are included to assess the confidence of protein identification:

- QValue: represents the FDR and provides an estimate of the proportion of incorrect identifications among accepted hits.
- PEP (Posterior Error Probability): estimates the likelihood that a given identification is incorrect, based on the posterior probability distribution of the match quality.

These scores, calculated at the protein group level, provide users with a transparent assessment of the reliability of each protein identification and serve as additional metrics for preprocessing data to ensure accurate analysis.

### Clinical data

The Clinical Data Table complements the proteomic dataset by capturing key demographic and clinical parameters for each subject. Recorded variables include age, sex, race, ocular diagnoses, visual acuity, intraocular pressure, medication usage, and other relevant ocular or systemic conditions. Each entry in the Clinical Data Table is linked to the corresponding record in the Protein Data Table through its ‘Sample ID’, which identifies a specific tear sample. Each entry also includes a ‘Subject ID’, which uniquely identifies the individual participant. This structure allows multiple samples to be associated with the same subject, e.g. samples collected from the left and right eyes at different timepoints or collected before and after treatment, while still maintaining a clear distinction between subject-level and sample-level information.

### Protein summary

**Figure 5.**
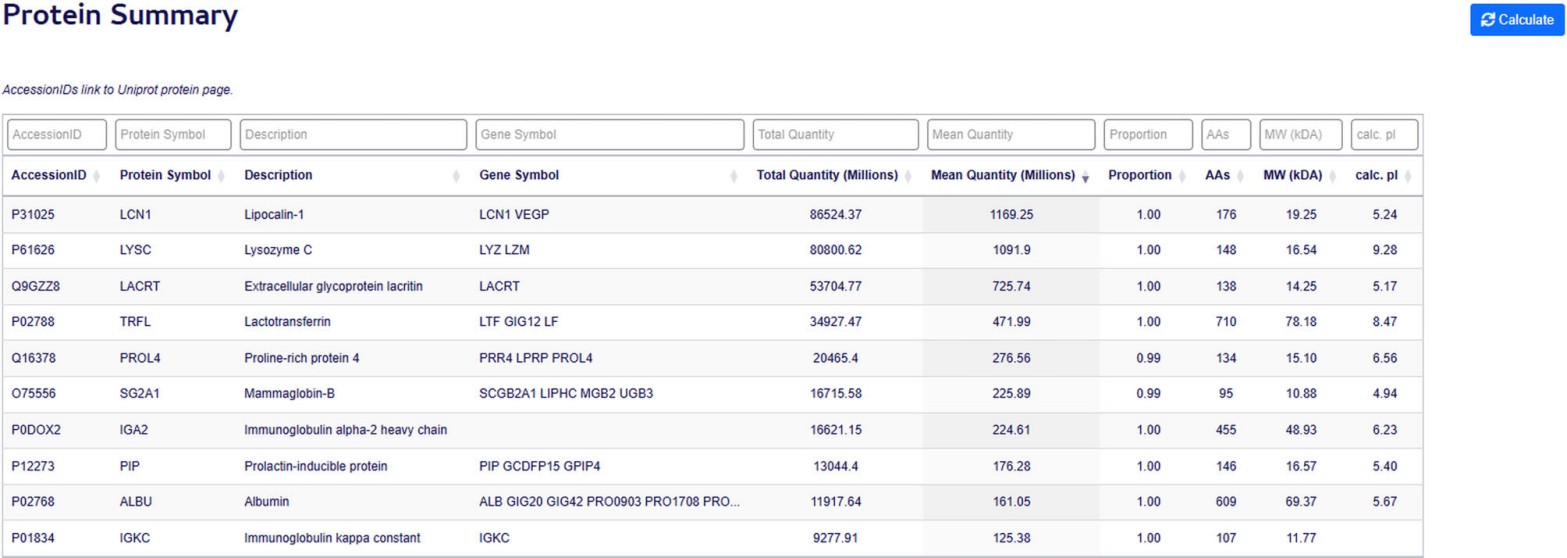
Protein summary page. The Protein Summary Table displays Accession ID, Protein Symbol, Protein Description, Gene Symbol, total and average protein abundance, Sample detection proportion, and molecular characteristics for each protein, including amino acid count, molecular weight, and calculated isoelectric point.

The Protein Summary Table provides a comprehensive overview of the proteomic landscape across all samples (Fig. 5). Each record corresponds to a unique protein, indexed by the ‘Accession ID’ field (equivalent to its UniProt identifier), which serves as the relational key linking to associated entries in the Protein Data Table. The table reports several key metrics for each protein:

- Total MaxLFQ Sum: the cumulative abundance of the protein across all samples.
- Average MaxLFQ: the mean abundance of the protein per sample.
- Detection frequency: the proportion of samples in which the protein was detected.

The proteins in the table are ranked in decreasing order based on their average MaxLFQ values. In addition to these quantitative metrics, the table provides detailed nomenclature and molecular attributes for each protein, including the full protein name, gene symbol, amino acid length, theoretical molecular weight, and predicted isoelectric point. Together, these features allow users to explore the structural and functional characteristics of the tear proteome alongside its abundance and prevalence.

### Most abundant proteins in tear fluid

The top 50 proteins detected in human tear fluid, ranked by average MaxLFQ intensity, are summarized in Table 2. These proteins encompass a broad range of biological functions that are critical for maintaining ocular surface homeostasis, particularly through antimicrobial and immune defence mechanisms. Key examples include lysozyme C (LYZ) and lactotransferrin (LTF), which mediate bacterial cell wall degradation and iron sequestration, respectively, and lipocalin-1 (LCN1), which limits microbial colonization and facilitates lipid transport. Immunoglobulin components such as IGHA1, IGKC, and JCHAIN further reinforce mucosal immunity by supporting antigen recognition and coordinated immune responses. Additional immune-modulatory proteins, including S100A8, S100A9, and SLPI, contribute to the regulation of inflammation and innate host defence.

**Table 2.** Top 50 most abundant proteins identified in human tear fluid.

Uniprot ID	Protein name	Gene	MaxLFQ (mil, avg ± SD)	Samples present (%)
P31025	Lipocalin-1	LCN1	1169.25 ± 1550.72	100.00
P61626	Lysozyme C	LYZ	1091.90 ± 1746.09	100.00
Q9GZZ8	Extracellular glycoprotein lacritin	LACRT	725.73 ± 1411.59	98.65
P02788	Lactotransferrin	LTF	471.99 ± 579.84	100.00
Q16378	Proline-rich protein 4	PRR4	276.52 ± 615.43	95.95
O75556	Mammaglobin-B	SCGB2A1	225.89 ± 460.63	98.65
P0DOX2	Immunoglobulin alpha-2 heavy chain		224.61 ± 251.75	100.00
P12273	Prolactin-inducible protein	PIP	176.28 ± 279.28	100.00
P02768	Albumin	ALB	161.05 ± 126.49	100.00
P01834	Immunoglobulin kappa constant	IGKC	125.38 ± 105.35	100.00
P01876	Immunoglobulin heavy constant alpha 1	IGHA1	115.57 ± 120.61	100.00
Q9UGM3	Scavenger receptor cysteine-rich domain-containing protein DMBT1	DMBT1	88.35 ± 156.43	100.00
P25311	Zinc-alpha-2-glycoprotein	AZGP1	69.85 ± 87.80	100.00
O95968	Secretoglobin family 1D member 1	SCGB1D1	61.85 ± 104.42	90.54
P05109	Protein S100-A8	S100A8	61.49 ± 35.27	100.00
A0M8Q6	Immunoglobulin lambda constant 7	IGLC7	53.80 ± 74.55	71.62
P01833	Polymeric immunoglobulin receptor	PIGR	39.74 ± 47.91	100.00
Q562R1	Beta-actin-like protein 2	ACTBL2	34.61 ± 16.19	100.00
P0DOX7	Immunoglobulin kappa light chain		31.88 ± 41.26	100.00
P04792	Heat shock protein beta-1	HSPB1	28.18 ± 17.90	100.00
P01036	Cystatin-S	CST4	26.38 ± 34.08	100.00
P68133	Actin, alpha skeletal muscle	ACTA1	25.79 ± 15.98	100.00
P0DOX5	Immunoglobulin gamma-1 heavy chain		24.59 ± 22.04	100.00
Q99935	Opiorphin prepropeptide	OPRPN	24.19 ± 32.46	100.00
P01591	Immunoglobulin J chain	JCHAIN	24.00 ± 25.86	100.00
P60709	Actin, cytoplasmic 1	ACTB	23.38 ± 14.19	100.00
P13646	Keratin, type I cytoskeletal 13	KRT13	22.60 ± 18.45	100.00
P06702	Protein S100-A9	S100A9	21.44 ± 15.27	100.00
P02814	Submaxillary gland androgen-regulated protein 3B	SMR3B	20.43 ± 53.76	79.73
P04406	Glyceraldehyde-3-phosphate dehydrogenase	GAPDH	20.02 ± 9.49	100.00
P09211	Glutathione S-transferase P	GSTP1	19.55 ± 9.30	100.00
P08727	Keratin, type I cytoskeletal 19	KRT19	19.01 ± 14.19	100.00
P04083	Annexin A1	ANXA1	18.94 ± 10.82	100.00
P01037	Cystatin-SN	CST1	15.95 ± 35.33	93.24
P03973	Antileukoproteinase	SLPI	15.92 ± 25.75	95.95
P13647	Keratin, type II cytoskeletal 5	KRT5	15.89 ± 15.30	100.00
Q99954	Submaxillary gland androgen-regulated protein 3A	SMR3A	15.71 ± 20.90	89.19
B9A064	Immunoglobulin lambda-like polypeptide 5	IGLL5	15.34 ± 21.48	64.86
P62805	Histone H4	H4C1	15.33 ± 13.09	100.00
P10909	Clusterin	CLU	14.10 ± 14.19	100.00
Q05639	Elongation factor 1-alpha 2	EEF1A2	13.38 ± 12.57	78.38
P21980	Protein-glutamine gamma-glutamyltransferase 2	TGM2	12.75 ± 7.21	100.00
P80188	Neutrophil gelatinase-associated lipocalin	LCN2	12.75 ± 17.22	100.00
P06733	Alpha-enolase	ENO1	12.66 ± 6.13	100.00
P04908	Histone H2A type 1-B/E	H2AC4	11.77 ± 12.28	78.38
P30044	Peroxiredoxin-5, mitochondrial	PRDX5	11.55 ± 5.94	100.00
Q06830	Peroxiredoxin-1	PRDX1	11.41 ± 5.51	100.00
P02787	Serotransferrin	TF	11.34 ± 8.52	100.00
P04264	Keratin, type II cytoskeletal 1	KRT1	10.98 ± 11.08	100.00
P19013	Keratin, type II cytoskeletal 4	KRT4	10.41 ± 7.51	100.00

Structural and cytoskeletal proteins, such as actins (ACTA1, ACTB) and multiple keratin isoforms, constitute another major functional group, reflecting their roles in maintaining epithelial cell architecture and preserving the ocular surface barrier. Protease inhibitors, including cystatin-S (CST4) and cystatin-SN (CST1), help prevent excessive proteolytic activity that could compromise tissue integrity. Proteins involved in molecular transport and lipid metabolism, such as albumin (ALB) and zinc-alpha-2-glycoprotein (AZGP1), support the movement of small molecules and contribute to tear film stability.

Enzymes and metabolic regulators, including GAPDH, GSTP1, and ENO1, indicate the presence of active metabolic and redox processes at the ocular surface. Stress-response proteins such as heat shock protein beta-1 (HSPB1) and clusterin (CLU) assist in protein folding and protect cells against oxidative damage. Finally, several signalling- and growth-associated proteins, including the tear-specific factor lacritin (LACRT) and proteins such as EEF1A2 and TGM2, participate in pathways related to epithelial homeostasis, protein synthesis, and tissue remodelling.

## Discussion

The TearFluid Database represents a significant advancement in ocular proteomics by establishing a centralized, publicly accessible, and scalable platform that integrates high-resolution protein abundance data with comprehensive clinical and demographic metadata. This resource addresses several unmet needs in tear-fluid proteomics and establishes normative reference ranges for tear-protein abundance, providing a valuable benchmark for comparative analyses across disease subtypes and clinical cohorts. Importantly, the integration of molecular and clinical data within a unified framework enables powerful multidimensional analyses that facilitate hypothesis generation, biomarker discovery, disease stratification, and systems-level investigations of tear-fluid biology. Overall, this platform serves as a foundational tool for advancing precision diagnostics and personalized therapeutics through the molecular analysis of tear-fluid samples.

The most abundant proteins identified in the dataset include lysozyme C, lactotransferrin, lipocalin-1, and lacritin. These proteins are well-established components of the tear film and play key roles in lipid transport [35], corneal epithelial cell regeneration [36], and antimicrobial and immunological defence [37, 38]. The consistent detection of these fundamental elements of the tear proteome validates our analytical approach and affirms the biological reliability of our findings. Notably, the identification of less-characterized proteins with high abundance or frequent detection highlights opportunities for novel biomarker discovery and hypothesis generation. These proteins may play underappreciated roles in tear-film homeostasis or ocular surface pathology and warrant further functional investigation.

The inclusion of parallel clinical metadata alongside proteomic profiles distinguishes the TearFluid Database from previously available repositories. By incorporating variables such as age, sex, race, ocular diagnoses, medication usage, and ocular surface parameters, the database enables users to explore biologically and clinically meaningful patterns across the dataset and derive new insights. This integrated structure supports stratified analyses across demographic and disease categories, facilitating the identification of subgroup-specific protein signatures and enhancing the biological interpretability of the findings.

The TearFluid Database platform was designed with scalability as a central priority. The use of an model-view-controller (MVC) architectural pattern enables efficient development and seamless expansion of the web application. Ongoing efforts in our laboratory are focused on increasing the dataset by analysing tear samples from a broad range of individuals with diverse ocular conditions, including healthy controls. Future work will involve collaborations with additional institutions to further expand the database. As more samples are processed, the resource will grow in both size and clinical diversity. This iterative expansion will enable the development of increasingly refined reference ranges and provide greater statistical power for subgroup analyses, thereby enhancing the platform’s utility for cross-sectional comparisons, longitudinal studies, and machine-learning applications.

Despite its strengths, the current version of the TearFluid Database has limitations that warrant consideration. The sample size, while larger than that of most existing tear proteomics studies employing high-resolution analyses, remains modest for population-level inference. As the database expands to include additional samples, particularly from diverse backgrounds and patients with specific ocular diseases, its statistical power and generalizability will improve. Additionally, although DIA-based proteomics offers high depth and reproducibility, it does not yet capture the full complexity of post-translational modifications, or protein isoforms, all of which are likely relevant to tear biology. Moreover, mass spectrometry-based proteomics cannot capture the full range of tear fluid proteins, particularly low-abundance proteins such as cytokines that are critical for ocular surface homeostasis, because they often exist below the detection limits of conventional mass spectrometry methods. Additionally, several proteins exhibit poor ionization efficiency, reducing their likelihood of detection. Future directions include expanding the database to incorporate longitudinal data, diseased cohorts (e.g. dry eye disease, glaucoma, autoimmune disorders), and cross-platform validation using complementary proteomic techniques, such as targeted MS or antibody-based methods.

## Summary

The Tear Fluid Database is a publicly accessible, scalable resource that integrates high-resolution DIA-based mass spectrometry data with comprehensive clinical and demographic metadata to advance tear fluid proteomics research. Built using an MVC architecture with ASP.NET, JavaScript, and Microsoft SQL Server, the platform provides an intuitive interface for querying, comparing, and exporting data across its Protein Data, Clinical Data, and Protein Summary modules. As one of the most extensive tear proteome resources currently available, it establishes normative reference ranges, supports cross-study comparisons, and enables researchers to perform discovery analyses, generate hypotheses, and uncover clinically meaningful patterns. This integrated framework provides a strong foundation for tear fluid biomarker discovery and translational research.

## Data Availability

Data are available for viewing and download at tearfluid.org.
